# Highly multiplexed single-cell quantitative PCR

**DOI:** 10.1371/journal.pone.0191601

**Published:** 2018-01-29

**Authors:** Michael VanInsberghe, Hans Zahn, Adam K. White, Oleh I. Petriv, Carl L. Hansen

**Affiliations:** Michael Smith Laboratories, University of British Columbia, Vancouver, British Columbia, Canada; University of Illinois at Chicago, UNITED STATES

## Abstract

We present a microfluidic device for rapid gene expression profiling in single cells using multiplexed quantitative polymerase chain reaction (qPCR). This device integrates all processing steps, including cell isolation and lysis, complementary DNA synthesis, pre-amplification, sample splitting, and measurement in twenty separate qPCR reactions. Each of these steps is performed in parallel on up to 200 single cells per run. Experiments performed on dilutions of purified RNA establish assay linearity over a dynamic range of at least 10^4^, a qPCR precision of 15%, and detection sensitivity down to a single cDNA molecule. We demonstrate the application of our device for rapid profiling of microRNA expression in single cells. Measurements performed on a panel of twenty miRNAs in two types of cells revealed clear cell-to-cell heterogeneity, with evidence of spontaneous differentiation manifested as distinct expression signatures. Highly multiplexed microfluidic RT-qPCR fills a gap in current capabilities for single-cell analysis, providing a rapid and cost-effective approach for profiling panels of marker genes, thereby complementing single-cell genomics methods that are best suited for global analysis and discovery. We expect this approach to enable new studies requiring fast, cost-effective, and precise measurements across hundreds of single cells.

## Introduction

Single-cell analysis preserves a wealth of information that is lost when measurements are instead taken by averaging cells together. While the importance of maintaining this resolution is well appreciated, techniques with the requisite sensitivity and scalability for single-cell molecular analysis have only recently been available. Perhaps the most significant advancement in this field is the development of technologies for measuring the variations in and expression of nucleic acids, the main thrust of which has been measurements of mRNA expression levels. This rapid advancement of evermore powerful measurement technologies has, in turn, spurred the development of new single-cell analytics that meet the unique challenges associated with interpreting large single-cell data sets [1, 2]. As a result, single-cell RNA expression profiling now provides new avenues for the classification of cell types, the identification of gene regulatory networks, and the high-resolution reconstruction of state transitions.

Single-cell measurements of transcription can generally be categorized as i) untargeted methods suitable for genome-wide discovery and profiling, or ii) targeted methods for assessing the expression of a panel of genes. A variety of genome-wide techniques have emerged over the past five years, each based on different methods of whole-transcriptome amplification and library construction, followed by high-throughput sequencing and bioinformatics-based quantification using read counts or binning of unique molecular identifiers [3–8]. Targeted transcript measurements are best suited to situations where a finite number of biomarkers of interest have already been established, and are generally faster, more sensitive, and less expensive than global studies. Methods for targeted analysis include reverse transcription polymerase chain reaction (RT-qPCR) [9–12] and single molecule counting using digital PCR [13, 14] or imaging of hybridization probes [15, 16]. Of these approaches, RT-qPCR is the most widely used and offers combined advantages of single-molecule sensitivity, speed to complete measurements in hours, and ease of assay design. The use of microfluidic arrays for the final qPCR step has been particularly useful for single-cell analysis, providing a means to greatly increase throughput and reduce costs [17–20]. This workflow, however, requires separate pre-processing steps for cell isolation, cDNA synthesis, and multiplexed pre-amplification to achieve the concentrations of cDNA that are needed for nanolitre-volume detection (S1 and S2 Tables).

Here we report the development of a microfluidic device that integrates the complete workflow for performing highly multiplexed RT-qPCR on up to 200 single cells. We first establish the technical performance on dilutions of purified RNA and validate on-chip cell processing by comparing our multiplexed measurements of widely used housekeeping genes to those made using existing single-cell qPCR solutions. We then apply our method to measuring miRNA expression in heterogeneous cell populations.

## Materials and methods

### Device fabrication

Microfluidic devices were fabricated using multilayer soft lithography [21]. Devices were designed using CAD software (AutoCAD, AutoDesk Inc.) and printed to 5-inch chrome photomasks (HTA Enterprises) using a LW405 mask writer (Microtech). All masks were printed with 8-μm resolution, except for the layer containing the cell traps, which was printed at 1-μm resolution. Photomasks were processed according to manufacturer directions. The CAD drawing of the device has been included in S1 File.

The set of master molds comprising one “flow” mold and one “control” mold were fabricated on 4-inch silicon wafers (Silicon Quest International). The control mold containing the valves and assay delivery channels was fabricated using a single layer of 25-μm high SU8-2025 (MicroChem). The flow mold was fabricated using six lithographic steps. First, an 8-μm high layer of Shipley’s Photoresist 220 (SPR220-7.0, DOW) was deposited to form the channels connecting the sample-splitting chambers to the detection chambers. These channels were rounded to enable valve closure by placing the processed wafer at 135°C for 10 minutes, then ramping to 190°C and holding for 2 hours. Next, 10-μm and 15-μm high layers of SU8-2010 (MicroChem) were successively deposited to form the sample-splitting and cell-capture chambers, respectively. A 15-μm high layer of AZ-50XT (AZ Electronic Materials) was then added to form the remaining valveable channels (between cell-capture sites, reagent delivery and general device control). This AZ layer was rounded by ramping the processed wafer from 65°C to 190°C in a convection oven, holding for 4 hours and slowly ramping to 25°C; the channel height after rounding was 13 μm. Finally, the reagent injection channels were formed using a 60-μm high layer of SU8-50 (MicroChem), followed by a 300-μm high layer of SU8-2150 (MicroChem) to form the chambers for reverse transcription, pre-amplification, and detection. All resist processing was performed according to manufacturer specifications. Master molds were coated with Parylene-C after fabrication [22] to facilitate subsequent elastomer release.

Microfluidic devices consisted of three layers of polydimethylsiloxane (PDMS RTV615, Momentive) elastomer, with a blank bottom layer, a middle layer containing the channels on the control mold, and then the flow layer on top. The flow layer was made by casting PDMS mixed at a 5:1 ratio (5 parts RTV615A to 1 part RTV615B) on the flow mold and baking at 80°C for 30 minutes. The thin control layer was made by spin-coating PDMS mixed at a 20:1 ratio at 1,800 rpm on to the control mold and immediately baking at 80°C for 20 minutes. After baking, both the flow and control layers were allowed to cool to room temperature. The flow layer was then peeled off of the mold and aligned to the spin-coated, baked control layer. Alignment was performed by hand under a dissection microscope progressing from one corner of the device to the other. As the feature array was too dense to allow for alignment marks, layer registration was performed using functional features. This structure was then baked at 80°C for 30 minutes to allow the two layers to bond. After bonding, the combined slab was peeled off of the control mold and access ports were punched using a coring tool (CR0350255N20R4, Syneo). Interlayer connections (vias) were then laser ablated using a custom instrument [23]. The blank layer was prepared by spin-coating 20:1 PDMS at 1,800 rpm on a blank wafer and baking this at 80°C for 20 minutes. The bonded, punched, and ablated control-flow slab was then placed onto the baked blank layer and allowed to bond at 80°C overnight. Finally, the combined 3-layer slab was peeled off the blank wafer, diced, and then bonded to glass slides using oxygen plasma (PDC-32G & PDC-FMG, Harrick Plasma). These mounted, finished chips were further cured at 80°C overnight prior to use.

Some experiments presented herein were done with an early prototype that contained 52 cell-processing units per subarray, for a total of 208 cells per device. Two cell processing units per subarray were removed from the final version in order to better accommodate the imaging area.

### Device operation

Microfluidic devices were operated semi-automatically using 21 solenoid actuators (Fluidigm Corp.) connected to a digital input-output card (NI PCI-DIO-32HS, NI PCI-6512, SCB-100, National Instruments) controlled using custom LabView (National Instruments) software. Teflon tubing fastened to 20-gauge hollow stainless steel pins (Small Parts Inc.), or gel-loading pipet tips were used to connect the chip to external pressure sources. Valves were operated at a pressure of 45 PSI, reagents were injected using 5 PSI, and cells were loaded at 1–2 PSI. Krytox 102 (DuPont) oil was used in the control lines isolating the qPCR detection chambers and water was used in the rest.

Lysis, reverse transcription, and pre-amplification thermal incubations were performed on a flatbed thermocycler block (Bio-Rad DNA Engine PTC-200; MJ Research/Bio-Rad) with light mineral oil (Fisher Scientific) added between the block and the glass slide.

Microfluidic quantitative PCR was performed on a prototype version of the BioMark Instrument, (Fluidigm) [12] with the following fluorescence imaging capabilities. Image resolution and bit depth: 4 megapixel, 16 bit. Filters: FAM: Ex 485/20 Em 525/25; VIC: Ex 530/20 Em 570/30; ROX: Ex 580/25 Em 610/15; QAS: Ex 580/25 Em 680/25. Light Source: 175 W xenon arc bulb.

### Cell loading

Cell-loading channels were first primed with 10 mg/mL bovine serum albumen (BSA, Gibco) in phosphate buffered saline (PBS, Gibco) [24]. Cells were then loaded in the device by applying 1–2 PSI to a gel loading pipet tip containing a single-cell suspension (1×10^5^–1×10^6^ cells/mL) in complete media. As cells flowed down the channel, they were captured at the start of each cell-processing unit by lithographically defined cell traps. After sufficient trap occupancy was obtained, cell wash buffer (1 mg/mL BSA in PBS) was flowed down the channel to remove untrapped cells, extracellular RNA, and debris. The cell isolation valves were then closed to partition the cell loading channel and isolate individual cell processing units. Each cell trap was then visually inspected using brightfield microscopy, and the status of each trap (single cell, multiple cells, debris, or empty) was recorded.

### Two-step RT-qPCR

Two-step RT-qPCR was used for all measurements made on microRNA. The workflow was based on those designed for multiplexed miRNA profiling on 100–1,000s of pooled cells [18] and single-plex measurements on single cells [12]. A chip-operation schematic for the two-step workflow is presented in S1 Fig.

Following visual inspection of the cell-capture chambers, the cells were lysed by placing the device onto a flatbed thermocycler and heating to 85°C for 7 minutes and then cooled to 4°C.

Complementary DNA (cDNA) synthesis was next performed using the High Capacity Reverse Transcription Kit (ABI). Gene-specific reverse transcription (RT) assays were first pooled to create a 5× working solution [2 μL of each of twenty 5× assays combined with 60 μL of TE buffer pH 8.0 (Ambion)]. Reverse transcription brew [1 μL 10× RT buffer, 2 μL 5× RT assay pool, 0.125 μL 100 mM dNTPs, 1.625 μL 50 U/μL MultiScribe reverse transcriptase, 0.0625 μL 20 U/μL RNase inhibitor, 0.2 μL 5% Tween-20 (Sigma Aldrich), brought up to 9.2 μL with UltraPure water (Gibco)] was flowed from the common reagent delivery bus over the cell traps and into the 10-nL reverse transcription chamber. Excess RT brew was washed out of the device by flowing 100 μL of reagent wash solution [0.1% Tween-20 in UltraPure water] through the reagent injection lines. The device was then placed on a flatbed thermocycler for a 2-minute incubation at 16°C, followed by 60 cycles of 2 minutes at 20°C, 30 seconds at 42°C, 1 second at 50°C, then finishing with a 5-minute incubation at 85°C, and cooled to 4°C.

Pre-amplification was next performed by adding pre-amplification brew [12.5 μL of 2× Universal Master Mix (Applied Biosystems), 2 μL 25 mM MgCl_2_ (Applied Biosystems), 1 μL 100 mM dNTP (Applied Biosystems), 1.25 μL 5 U/μL AmpliTaq Gold (Applied Biosystems), 1.25 μL 20× pre-amplification primer pool, 0.5 μL 5% Tween-20, brought up to 20 μL with UltraPure water] through the common reagent injection line, diluting the cDNA product into the 50-nL pre-amplification chamber. After completely filling this chamber, the excess pre-amplification brew was then washed out of the device by flowing 100 μL of wash solution through the reagent injection lines. The device was transferred to a thermocycler and incubated at 25°C for 15 minutes to ensure complete mixing of cDNA product with the pre-amplification brew. The device was then incubated at 95°C for 10 minutes, 55°C for 2 minutes, then six cycles of 72°C for 2 minutes, 95°C for 15 seconds, and 60°C for 4 minutes. The device was then incubated at 99°C for 10 minutes and then cooled to 25°C. The 20× pre-amplification primer pool contained 3 μL of each 20× assay brought to a final volume of 100 μL with TE pH 8.

Following pre-amplification, the reagent injection lines were again washed with 100 μL of wash solution, which was then used to push the pre-amplification product into the 0.15-nL sample splitting chambers. Valves were then actuated to isolate the sample splitting chambers. The independent qPCR brews [12.5 μL 2× Universal Master Mix no UNG (Applied Biosystems), 1.25 μL 20× TaqMan qPCR assay, 2.5 μL 1% Tween-20, 0.25 μL 40× ROX Reference Dye (Invitrogen), brought up to 24.42 μL with UltraPure water] were then used to push the metered pre-amplification product into the 6.4-nL detection chambers. The device was then transferred to an instrument for microfluidic real-time PCR and thermocycled with the following conditions: 95°C for 10 minutes, then 40 cycles of 95°C for 15 seconds, 60°C for 1 minute and fluorescence imaging at 60°C.

### One-step RT-qPCR

One-step RT-qPCR was used for all measurements made on mRNA and was performed using the CellsDirect kit (Invitrogen). Processing steps are similar to those used for the two-step workflow, with the main distinctions being that the “RT chamber” was instead used for cell lysis, and the “pre-amplification chamber” was used for both cDNA synthesis and pre-amplification steps. A chip-operation schematic for the one-step workflow is presented in S2 Fig.

Following visual inspection of the cell-capture chambers, the cells were lysed by flowing lysis buffer [10 μL Lysis Resuspension Buffer (Invitrogen), 1 μL Lysis Enhancer Solution (Invitrogen)] over the cell traps into the 10-nL “RT chamber”. Excess lysis buffer was washed out of the reagent injection line with 100 μL of wash solution. The device was then incubated at 25°C for 10 minutes, then 70°C for 10 minutes and cooled to 4°C.

cDNA synthesis and pre-amplification were then performed by pushing the lysate into the 50-nL pre-amplification chamber with the one-step RT-PCR brew (12.5 μL 2× Reaction Mix with ROX, 0.5 μL 50 mM MgSO_4_, 1.67 μL 15× pooled primer mix, 0.5 μL Superscript III/Platinum Taq enzyme mix, 2.5 μL 1% Tween-20, brought to a final volume of 19.62 μL with UltraPure water). The 15× pooled primer mix contained 1.5 μL of each 20× TaqMan assay, brought to a final volume of 40 μL with TE pH 8.0. The excess brew was then washed out of the device with 100 μL of wash solution. The device was then transferred to a thermocycler and incubated at 25°C for 15 minutes for diffusive mixing, then at 50°C for 20 minutes for cDNA synthesis, followed immediately by 95°C for 2 minutes, then 12 cycles of 95°C for 15 seconds and 60°C for 4 minutes. The device was then incubated at 99°C for 10 minutes and then cooled to 25°C.

Following cDNA synthesis and pre-amplification, the reagent injection lines were again washed with 100 μL of wash solution, and this was then used to push the pre-amplification product into the 0.15-nL sample splitting chambers. Valves were then actuated to isolate the sample splitting chambers. The independent qPCR brews [12.5 μL 2× Universal Master Mix no UNG (Applied Biosystems), 1.25 μL 20× TaqMan assay, 2.5 μL 1% Tween-20] were then used to push the pre-amplification product into the 6.4-nL detection chambers. Quantitative PCR was then performed with the following conditions: 95°C for 10 minutes, then 40 cycles of 95°C for 15 seconds, 60°C for 1 minute and fluorescence imaging at 60°C.

The following assays were used for mRNA detection: *GAPDH* Hs02758991_g1 (ABI); *TBP* Hs00427620_m1 (ABI); *GGCX* Hs00168139_m1 (ABI); *BCR-ABL* Hs01036528_m1 (ABI); and *RPPH1* forward GAGGTCAGACTGGGCAGGAG, reverse CCTCACCTCAGCCATTGAACTC, probe FAM-TGCCGTGGACCCCGCCCTTCG-BHQ1 (Integrated DNA Technologies) [25].

### Cell culture and RNA purification

K562 cells were obtained from ATCC (CCL243) and were cultured in DMEM (Gibco) supplemented with 10% FBS (Gibco) and 1× GlutaMAX (Gibco). IL-3 independent BaF3 cells were a generous gift from Dr. James Piret at the University of British Columbia. They were cultured in RPMI-1640 (Gibco) supplemented with 10% FBS and 1× GlutaMAX. K562 cells had an average diameter of 16.8 ± 1.3 μm, and BaF3 cells had an average diameter of 13.5 ± 1.9 μm.

Purified total RNA was extracted from K562 cells using the mirVana RNA isolation kit (Ambion) according to manufacturer directions, omitting the optional size-enrichment step.

### Data analysis and plotting

Data are reported as mean ± standard deviation (SD). Fluorescence images were analyzed using scripts written in MATLAB (MathWorks, version 2014b) to extract real-time PCR curves and algorithmically calculate cycle-threshold (CT) values [12]. All subsequent data analysis was performed using R (version 3.4.0) with plyr (v1.8.4) and reshape (v0.8.6) packages. Plots were generated using ggplot2 (v2.2.1), ggbeeswarm (v0.6.0), cowplot (v0.7.0), gplots (v3.0.1), corrgram (v1.12), and RColorBrewer (v1.1–2). Values for boxplots were calculated using the default settings in geom_boxplot (ggplot2): the middle line represents the median, the lower and upper hinges correspond to the first and third quartiles, the upper whisker extends from the upper hinge to the largest value no further than 1.5 times the interquartile range, the lower whisker extends from the lower hinge to the smallest value no further than 1.5 times the interquartile range, and data beyond the whiskers are deemed outliers and plotted individually. Data have been deposited in the NCBI Gene Expression Omnibus under accession GSE102734. Details for specific sections are reported below.

### Single-molecule sensitivity

Total RNA at limiting dilution was analyzed using the one-step RT-qPCR workflow with 12 cycles of pre-amplification and assayed against *GAPDH*. A single-molecule CT cut-off was calculated to contain 99% of the distribution of CT values from cell-processing units that generated a signal in all twenty detection chambers (mean CT of 21.5 added to 2.5758 multiplied by the standard deviation of 0.67). Cell processing units were defined to have a successful single-molecule amplification event if greater than 75% of the detection chambers had a CT value lower than this cut-off.

The digital array response curve [26] for 52 chambers was used to convert the number of positive cell-processing units per array to the expected number of input molecules and corresponding 95% confidence intervals (S3 Table).

### Expression analysis

Amplification efficiency was measured based on the slope of a linear least-squares fit of the log of the total RNA concentration versus cycle threshold values. Data were pooled from three separate device runs, and the inverse of the standard deviations of the measurements at each concentration were used as the fit weights. Efficiency was calculated as $100\%\times (1-10^{-\frac{1}{slope}})$. Efficiency uncertainties are reported as a range based on the standard error (SE) from the least-squares fit.

Cycle threshold values were converted to copy number based on our measurements of the *GAPDH* single-molecule CT and amplification efficiency, namely $Copy\;number\;=\;2^{{CT}_{single\;molecule}-\;{CT}_{measured}}$. For miRNA analysis, five cycles were added to the *GAPDH* cut-off to account for the difference in pre-amplification PCR.

Differential miRNA expression between K562 and BaF3 cells was assessed using a Wilcoxon rank-sum test. P-value adjustments for multiple testing were computed using the Benjamini-Hochberg correction, and significance was determined at a false discovery rate (FDR) of less than 0.01. Hierarchical clustering was performed on log-scaled expression values using (1-pearson correlation)/2 as a distance metric and ward.D2 linkages.

Measurement precision was calculated as the standard deviation σ divided by the mean μ for the copy-number normalized measurements on purified RNA at the specified concentration; i.e. $Precision\;=\;100\%\;\times \;\frac{\sigma_{200\;pg}}{\mu_{200\;pg}}$.

### Correlation analysis

Expression correlations between transcripts were computed using Spearman’s rho. The significance of a correlation coefficient was assessed based on a null distribution derived by recalculating the correlation coefficient for randomly permuted single-cell expression measurements. Correction for multiple testing across multiple pairs was controlled using a Benjamini-Hochberg correction. Significance was assessed at an FDR of 0.01.

## Results and discussion

### Device design and operation

We designed a microfluidic device that integrates and parallelizes the processing steps necessary for multiplexed gene expression analysis, from cell capture through to qPCR. The resulting architecture uses several design elements from our previous systems for simplex quantitative and digital PCR [12, 14], including cell traps and sequential reaction chambers. In contrast to these implementations, however, the reaction products from each cell are split across twenty independently addressable reaction chambers for the final qPCR readout. An important consideration during the design process was to maintain measurement sensitivity during this multiplexed analysis. Even under ideal conditions, splitting the contents of a single cell across several different reactions severely limits the detection sensitivity. Therefore, a key element of our design addresses this potential shortcoming with the inclusion of fluidics that allow for a low-cycle multiplexed pre-amplification step prior to simplex qPCR.

**Fig 1:**
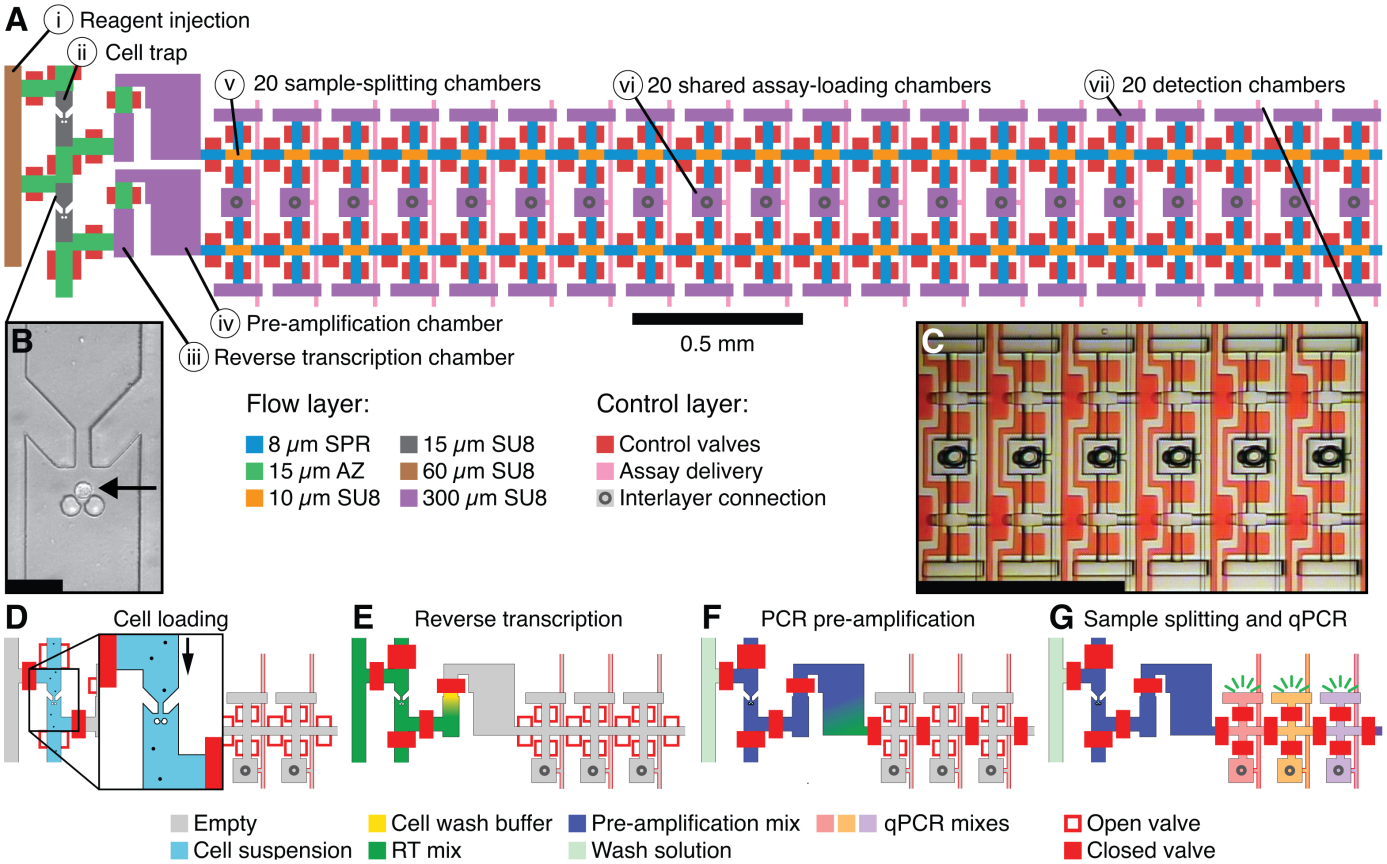
Multiplex RT-qPCR device schematic and operation. (A) Two cell-processing units. Components include (i) a reagent injection bus, (ii) a 0.3-nL cell capture chamber, (iii) a 10-nL reverse transcription (RT) chamber, (iv) a 50-nL pre-amplification chamber, (v) twenty 0.15-nL sample-splitting chambers, (vi) twenty shared assay-delivery chambers, and (vii) twenty 6.4-nL detection chambers. The “flow” layer is made up of features of six different heights. Control valves and assay-delivery channels are on the same 25 μm high SU8 “control” layer. Assay delivery from the control layer to the flow layer occurs through laser-ablated interlayer connections. Scale bar 0.5 mm. (B) Optical micrograph of a single K562 cell (indicated by a black arrow) caught in a cell trap. Scale bar 50 μm. (C) Optical micrograph of a subsection of the detection array. Control valves are coloured red. Scale bar 0.5 mm. (D-G) Schematic illustration of device operation. (D) Single cells are first loaded into the device, then washed, isolated, and lysed *in situ*. (E) Reverse transcription brew is then injected into the RT chamber, mixed with the lysate, and then the device is thermocycled. (F) Similarly, multiplexed pre-amplification mix is injected into the device, mixed with cDNA, and then the device is again thermocycled. (G) Finally, the pre-amplification product is split between detection chambers for qPCR.

The device features four linear arrays of 50 cell-processing units for a total capacity of up to 200 cells analyzed per device (S3 Fig). Each cell-processing unit (Fig 1A) is comprised of i) a reagent injection channel, ii) a 0.3-nL cell capture chamber, iii) a 10-nL reverse transcription (RT) chamber, iv) a 50-nL PCR pre-amplification chamber, v) twenty 0.15-nL sample splitting chambers, vi) twenty shared assay loading chambers, and vii) twenty 6.4-nL qPCR detection chambers. During operation, reactions are assembled in parallel, with reaction brews transferring and mixing with previously generated intermediates in the next reaction chamber. Sequential reagent metering is achieved by dead-end filling lithographically defined volumes, causing trapped air to be expelled into the gas-permeable PDMS elastomer from which the device is made. The reaction chambers were configured to provide sufficient dilution between each processing step so as to avoid reaction inhibition, and in such a way to accommodate a variety of assay types, including one- and two-step RT-PCR workflows.

In performing a two-step RT-PCR workflow (S1 Fig), device operation first begins with passivation of the cell-capture chambers to avoid cell adhesion during loading. Next, a single-cell suspension is injected into the device and flows through channels that have lithographically defined cell traps designed to isolate and capture cells at the front of each cell processing unit (Fig 1B and 1D). Following cell trapping, the cell-loading channel is flushed with cell wash buffer to expel any untrapped cells and extracellular RNA. The trapped cells are then isolated by actuation of separation valves, and the device is placed on a thermocycler to perform heat lysis. Reverse transcription brew is then injected through the cell-capture chamber into the neighbouring RT chamber, and allowed to diffusively mix with cell lysate (Fig 1E). The RT chamber is then isolated, and the device is again placed on a thermocycler for temperature control during cDNA synthesis. Next, the reagent injection channel is flushed with pre-amplification brew, and injected through the cell-capture and RT chambers, thereby pushing cDNA into the pre-amplification chamber (Fig 1F). The pre-amplification chamber is then isolated, and the device is transferred to a thermocycler for low-cycle PCR pre-amplification of all targets using multiplexed primer sets. On completion of the pre-amplification, ~20% of the resulting product is pushed into the sample-splitting chambers. Each individual gene-specific qPCR assay brew is then injected into a shared assay-loading chamber, through each isolated sample-metering chamber, and into each of the qPCR detection chambers (Fig 1G). These detection chambers are then isolated and the device is placed on a thermocycler with fluorescence imaging capabilities to perform qPCR. Finally, custom image processing software is used to extract the fluorescence signal in each chamber during PCR cycling, generate characteristic amplification curves for qPCR-based quantification, and calculate cycle threshold (CT) values.

Device operation for performing one-step RT-PCR (S2 Fig) closely follows the two-step protocol, except that a non-denaturing lysis buffer is flowed over the trapped, washed, and isolated cells into the “RT chamber”, and one-step RT-PCR brew is then used to push the cell lysate into the “pre-amplification chamber” to perform both reverse transcription and PCR pre-amplification.

A single device run consists of 4,000 qPCR reactions, can easily be completed within a day, and, with optical multiplexing, can produce upwards of 8,000 single-cell measurements. We note that implementing multistep processing at this scale required the fabrication of devices with very dense fluidic integration; each device integrates roughly 15,000 valves and 2,800 layer interconnects (vias) within a total footprint of 3.5 × 5 cm (S3 Fig). To achieve this reaction density we combined several advancements: high aspect ratio lithography (> 5:1) to increase reactor volumes using minimal device area, a 64% reduction in the standard valve size that is used in multilayer soft lithography (MSL) [21], and the use of three-dimensional microfluidic fabrication techniques [23] to support complex fluid routing.

### Device characterization

To benchmark the performance of our device we assessed assay linearity, precision, and sensitivity under different workflows and using assays of varying complexity. In order to remove the contributions of cell-to-cell variability, these measurements were done using dilution series of purified total RNA obtained from K562 cells, a human *BCR/ABL1* positive cell line from a patient with chronic myeloid leukemia [27]. Input RNA concentrations were varied from 200 to 0.2 pg per cell-processing unit, corresponding to approximately 10 to 1/100 cell-equivalents, assuming 20 pg of total RNA per cell [12].

**Fig 2:**
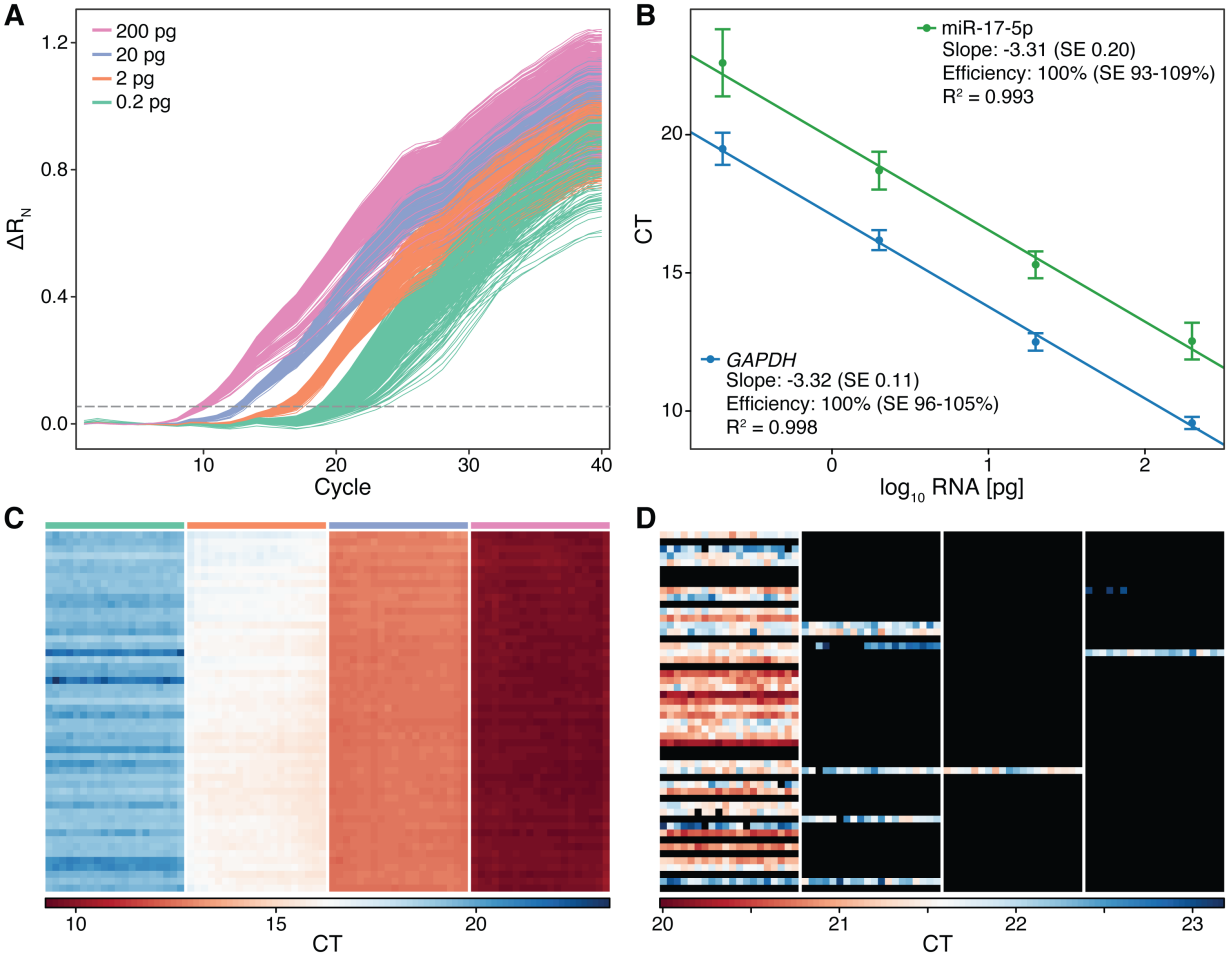
Device characterization and performance. (A) 4,160 real-time amplification curves generated from one experiment on a 10× dilution series of purified total K562 RNA. Each curve is generated from processing fluorescence images of the entire device taken after each cycle of PCR. The threshold for determining CT values is indicated by the dashed line. (B) Standard curves derived from three replicate 10× dilution series of K562 RNA that validate the one- and two-step workflows measured using *GAPDH* (blue) and miR-17-5p (green), respectively. Points represent the mean ± standard deviation of all 1040 detection chambers per concentration from all three replicates. Input RNA ranged from 200 pg (10 cell equivalent) to 0.2 pg (1/100 cell equivalent) per cell processing unit. (C) Heatmap representation of the CT values extracted from the real-time curves shown in A. Values are organized according to the device layout; the leftmost panel corresponds to the lowest concentration, the rightmost panel corresponds to the highest concentration and each row within each panel originates from the same cell-processing unit. The three rightmost panels (200–2 pg/cell processing unit) indicate uniform amplification across the device. The variability within the leftmost panel (0.2 pg/cell processing unit) is much higher than that within each cell-processing unit (row), illustrating the increased contribution of stochastic sampling towards the variability of this measurement. (D) Heatmap of CT values for *GAPDH* measured on a 5× dilution series at limiting RNA concentrations. Values are organized according to device layout, with the highest concentration seen on the left. Black cells were not detected. After sufficient PCR pre-amplification, cell-processing units that initially contained a single cDNA molecule were amplified to levels such that signal is produced in an average of 19.8/20 detection chambers.

We first tested assay linearity and amplification uniformity using a simplex assay for the expression of *GAPDH*, a widely expressed gene that is often used as an endogenous control. A *GAPDH* hydrolysis probe assay and 12 cycles of specific target pre-amplification was used with the one-step workflow for RT-qPCR (Fig 2A). The efficiency of amplification, as determined by the slope of the linear least-squares fit of log total RNA concentration versus CT, was measured to be 100% (SE: 96 to 105%) with an R^2^ of 0.998 (Fig 2B). The CT values obtained across all replicates ranged from 9.57 ± 0.22 to 19.49 ± 0.58, indicating uniform amplification across the array (Fig 2C), and a measurement precision at the higher concentrations of approximately 14.5%, approaching the limit of qPCR. While the technical variability increased at lower concentrations, this noise can be attributed to stochastic sampling in the initial RNA partitioning, as the variability within each cell processing unit was significantly lower than the variability between cell processing units (Fig 2B and 2C, S4 Fig) (e.g. standard deviation of the CTs from the lowest concentration was 0.58 ± 0.05, whereas the average standard deviation of CTs from each cell processing unit was significantly different at 0.18 ± 0.07, p = 0.0034, Wilcoxon rank-sum test).

We next assessed these same performance metrics under demanding multiplexed assay conditions. A two-step workflow was used with a pool of 20 commercially available stem-loop microRNA assays [28] for gene-specific cDNA synthesis and 7 cycles of pre-amplification (let-7b-5p, let-7c-5p, let-7d-5p, miR-10a-5p, 150-5p, 155-5p, 16-5p, 17-5p, 17-3p, 181a-5p, 200c-3p, 20a-5p, 221-3p, 223-3p, 24-3p, 27a-3p, 29b-3p, 451, 93-5p and 184-3p). In order to measure amplification uniformity across the device, these multiplexed reaction intermediates were all assayed against one of the highly expressed microRNA from this pool (miR-17-5p). Based on three independent device replicates, the amplification efficiency was again measured to be 100% (SE: 93 to 109%) with an R^2^ of 0.993 (Fig 2B). The CT values from all replicates ranged from 12.5 ± 0.6 to 22.6 ± 1.2, corresponding to a precision at the higher concentrations of approximately 31.5%.

We next characterized the detection sensitivity by measuring RNA at limiting dilution. In the absence of amplification prior to detection, single cDNA molecules would only be sporadically seen in individual detection chambers of positive cell-processing units. However, with pre-amplification, ideally enough product is generated from a single cDNA molecule to result in the above-background qPCR amplification in all twenty detection chambers. Four concentrations of purified K562 RNA at limiting dilution were mixed with one-step RT-qPCR brew, each loaded into one of the linear arrays, processed according to the one-step workflow, and assayed against *GAPDH*. The resulting pattern of CT values across the device had several characteristics indicative of single-molecule amplification (Fig 2D, S5 Fig). First, there was clear separation in the chip-wide distribution of CT values between single cDNA molecules and non-specific background (S5 Fig). The average single-molecule CT was found to be 21.4 ± 0.6, corresponding to a cut-off CT (capturing 99% of the distribution) of 23.2. Second, using this cut-off, positive detection chambers were organized based on their respective cell-processing unit (Fig 2D, amplification is seen across rows). In these positive cell-processing units, an average of 19.8 out of 20 detection chambers showed amplification, whereas only 0.1 out of 20 amplified in negative units. Finally, the observed dilutions between successive linear arrays were found to be in agreement with shot-noise resulting from a Poisson distribution of starting template molecules (S3 Table). Together, these results demonstrate a lower sensitivity limit of a single cDNA molecule. We note that successful detection, however, depends on conversion from RNA to cDNA, a process that has been previously seen to be variable between templates, assays, and enzymes [29].

**Fig 3:**
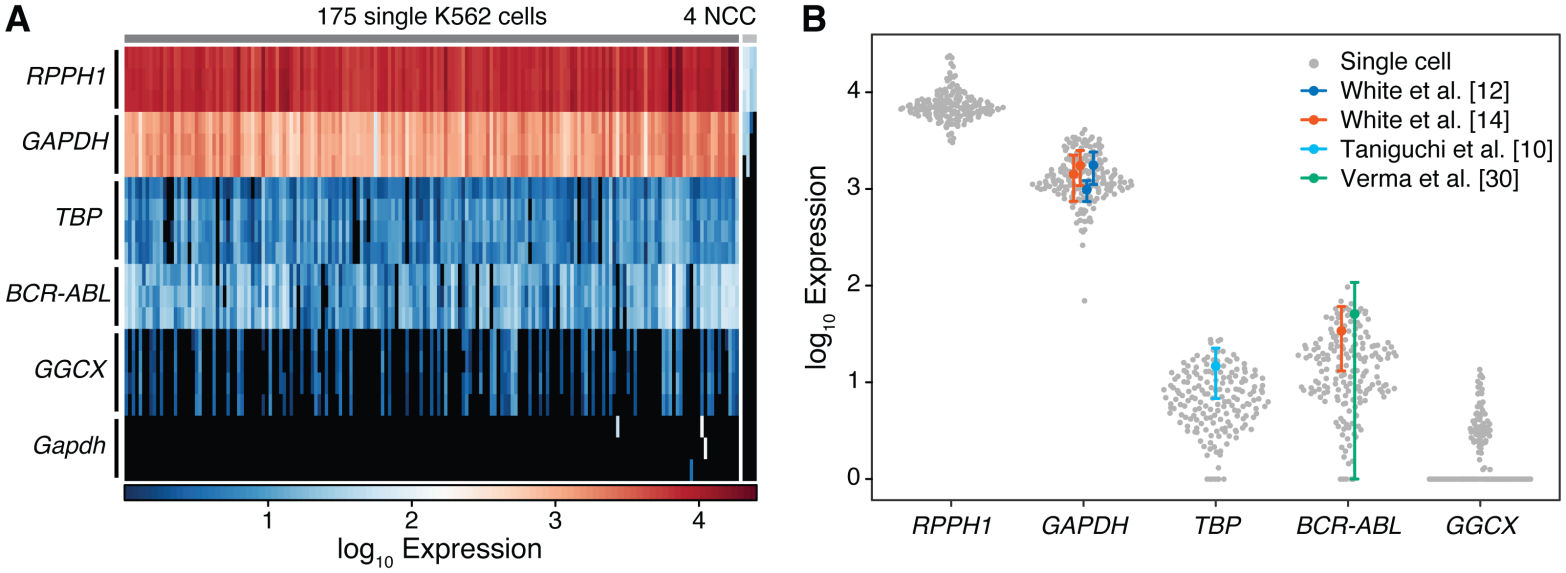
Single cell mRNA expression. (A) Heatmap of expression values of six mRNA on 175 single K562 cells and 4 no cell controls (NCC). Each column represents a cell, and each row represents an assay. Replicate measurements are grouped according to assay. As expected, murine *Gapdh* is not detected, only spuriously generating signal in 4/525 detection chambers. No-cell controls are clearly distinguishable from those from single cells, with an average difference in magnitude greater than 100×. The variability between replicate measurements for each gene is much smaller than the variance in expression seen between different cells. (B) Distribution of the copy number of each gene measured in each single cell, along with the mean expression values obtained from previously published single-cell qPCR measurements [10, 12, 14, 30]. Error bars represent the mean ± standard deviation.

We next evaluated the performance of on-chip cell processing by measuring gene expression in 175 single K562 cells. These tests were performed using an assay panel designed to interrogate genes that spanned a wide range of abundance and included i) three genes typically used as endogenous controls in bulk gene expression experiments (human *GAPDH*, *RPPH1*, and *TBP*), ii) a fusion transcript associated with CML (*BCR-ABL*), iii) a heterogeneously expressed gene (*GGCX*), and iv) a negative control (murine *Gapdh*). Using the previously derived single-molecule CT cut-off, and requiring positive amplification in all replicates for each cell, we detected both *RPPH1* and *GAPDH* in 100% (N = 175), *TBP* in 92%, *BCR-ABL* in 92%, and *GGCX* in 31.4% of single cells (Fig 3A). As expected, murine *Gapdh* was not detected in the vast majority of reactions, with only 4 out of 525 detection chambers generating signal, and these hits were spread across different cell processing units. While we initially expected both *TBP* and *BCR-ABL* to be ubiquitously expressed, six of the cells (3.4%) failed to generate signal in all replicates for each gene. This repeated absence suggests that these cells are true negatives, which, consistent with the low copy-number of these transcripts, might be explained by stochastic expression in some cells. Adjacent processing units that did not contain a cell (no cell controls, NCC) were clearly separated from single-cell measurements (Fig 3A). In these control samples, *TBP*, *BCR-ABL*, and *GGCX* all showed no amplification. *RPPH1* and *GAPDH* had an average difference of 154 and 116 fold, respectively, showing that cross-contamination arising between cells or due to free nucleic acid contamination is less than 1 part in 100. Finally, similar to the effects of stochastic sampling seen between measurements of low concentrations of purified RNA, the measurement variability between replicates was substantially smaller than the expression variability seen between single cells (S6 Fig).

As expected, these genes were expressed with a wide array of abundances spanning approximately four orders of magnitude (Fig 3B). The average number of cDNA molecules per cell were 7,734 (SD = 3,284) for *RPPH1*, 1,488 (SD = 815) for *GAPDH*, 7.2 (SD = 5.5) for *TBP*, 17.7 (SD = 16.2) for *BCR-ABL* and 1.4 (SD = 2.4) for *GGCX*. For *GAPDH*, these results closely match our earlier validation experiments on purified K562 RNA, which had an average of 1,730 (SD = 387) cDNA molecules per single-cell equivalent (20 pg) (cells: mean CT 12.91, SD = 0.84, purified RNA: mean CT 12.50, SD = 0.32) and are also consistent with our previously published estimates of *GAPDH* copy-number in single K562 cells [979 (SD = 240) to 1,761 (SD = 649) copies per cell, N = 233 and 1,421 (SD = 599) to 1,741 (SD = 573) copies per cell, N = 273 for references [12, 14], respectively]. Similarly, our results on *BCR-ABL* agree with previous measurements done by us [33 (SD = 18.9) copies per K562 cell, N = 242, ref. [14]) and Verma et al. [49.9 (SD = 57.2) copies per K562 cell, N = 69, ref. [30]), and those on *TBP* correspond with an independent measurement of 13.7 (SD = 7.9) copies per HCT116 cell, N = 14 [10]. We note that there was only a modest correlation in expression between the endogenous control genes (average spearman correlation 0.51, SD = 0.17, S7 Fig), suggesting that their expression is not simply a reflection of cell size and indicates the presence of additional sources of variability. This observation further reiterates previous recommendations against using traditional reference genes in normalizing single-cell expression data [13, 14, 31].

Together, these multiplexed RT-qPCR gene expression measurements on single cells are all in close agreement with both the quantities and variabilities obtained in previous studies. Combined with our initial validation experiments on purified RNA, they demonstrate the requisite precision, sensitivity, and specificity over the dynamic range necessary for single-cell analysis.

### Measurement of single-cell miRNA expression

MicroRNAs are a class of small (~22 nt) non-coding RNA that direct the RNA-induced silencing complex to post-transcriptionally degrade, destabilize, or repress mRNAs [32]. They are involved in a vast array of biological processes including development [33] and oncogenesis [34], and cancer-type classifiers based on their expression profiles have been shown to outperform those based on mRNA expression [35]. Owing to these characteristics, single-cell measurements of miRNAs may offer high potential for dissecting cell type and origin within heterogeneous populations.

To evaluate our device for performing single-cell miRNA profiling, we measured the expression of a panel of miRNAs on two distinct hematopoietic cell lines, K562 and BaF3. K562 is an undifferentiated human erythroleukemic cell line [27] that has been used extensively as a benchmark for single-cell genomics and BaF3 is a mouse pro-B-cell line [36]. The motivation for using cell lines of related lineage, but from two different species, was to assess potential evolutionary conservation of miRNA regulation pathways. Furthermore, as these cell lines are both derived from hematopoietic progenitor cells with different lineage potential and are known to spontaneously differentiate, they also provide inherently heterogeneous populations. Profiling was performed against twenty conserved miRNAs (let-7b-5p, miR-10a-5p, 150-5p, 155-5p, 16-5p, 17-5p, 17-3p, 181a-5p, 200c-3p, 20a-5p, 221-3p, 223-3p, 23a-3p, 24-3p, 27a-3p, 29b-3p, 451, 92a-3p, 93-5p, and snoRNA-142) that were selected to capture this extrinsic and intrinsic heterogeneity and include miRNAs previously associated with hematopoietic development [18, 37, 38].

**Fig 4:**
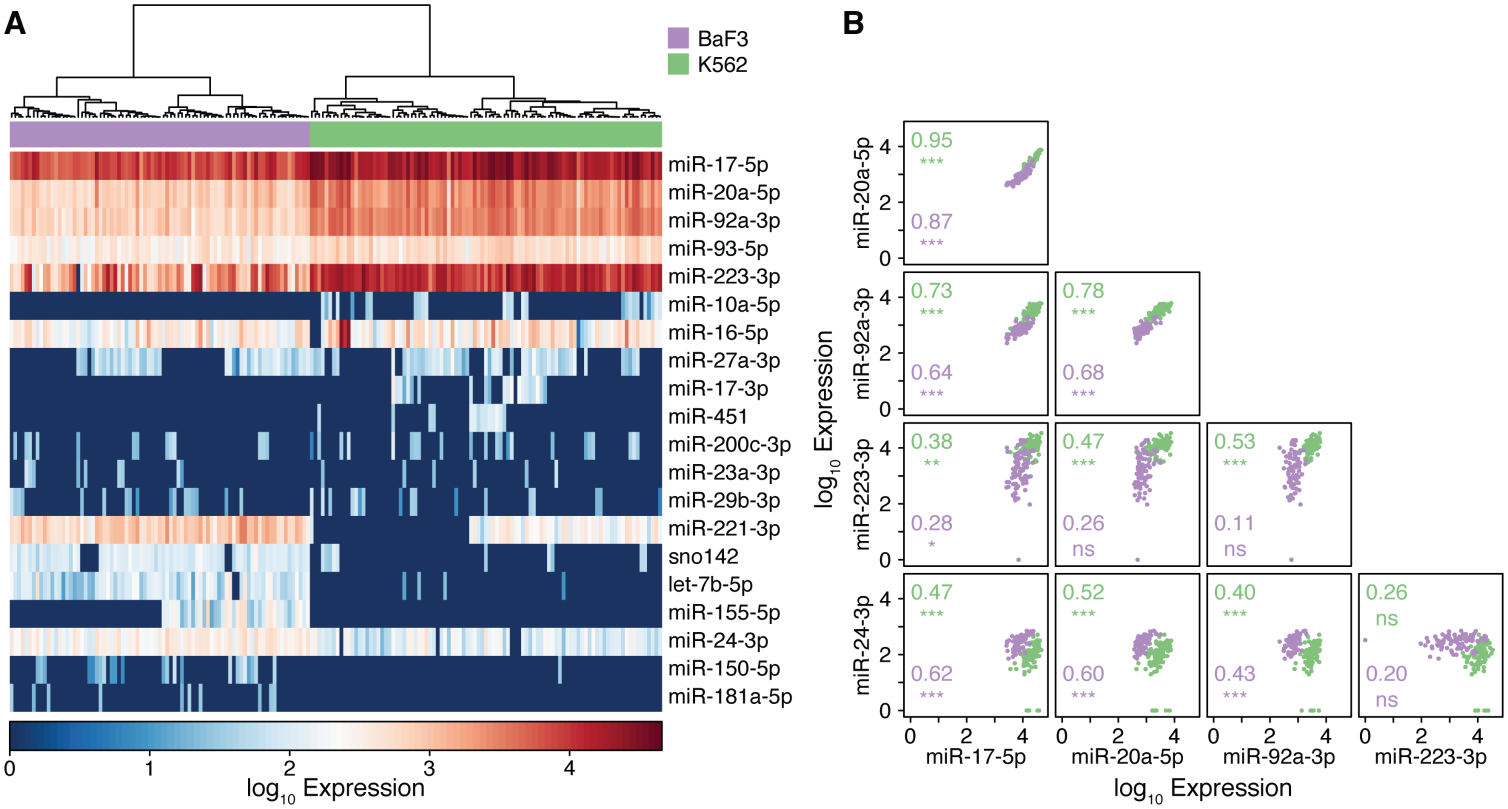
Single-cell miRNA expression. (A) Heatmap of expression values of 20 miRNAs in each of 95 K562 cells (green) and 81 BaF3 cells (purple). Unsupervised hierarchical clustering correctly groups the cell types based on their miRNA expression signatures. (B) Scatterplots of co-expressed miRNAs. miRNAs 17-5p, 20a-5p and 92a-3p are all strongly co-expressed as they are members of the miR-17~92 polycistronic cluster. The co-expression of miRNA 24-3p with these three miRNAs demonstrates how single-cell resolution identifies their positive regulation where bulk measurements would predict their negative regulation. Spearman correlation coefficients and their co-expression significance are denoted for each pair; * *p* < 0.05, ** *p* < 0.01, *** *p* < 0.001, ns not significant.

Fig 4A shows results from a single device run in which half of the array was loaded with K562 cells and half with BaF3 cells. Loading resulted in a total of 95 single K562 cells and 81 single BaF3 cells, as determined by bright-field microscopy, for a total single-cell occupancy of 85%. MicroRNAs were found to be expressed across a wide dynamic range, with miR-17-5p detected in 100% of the analyzed cells at an average of 15,700 (SD = 9,900) copies per cell, and miR-181a-5p detected in 5.7% of the cells at an average of 1.5 (SD = 6.5) copies per cell. As expected, a substantial proportion (15/20, 75%) of the analyzed miRNAs were found to be significantly differentially expressed between the two cell populations (S8 Fig). However, it is notable that, despite the obvious biological differences between these two cell types, only a minority of the miRNAs (7/20, 35%) exhibited strict cell-type expression specificity, and that these specific miRNAs were generally those that were weakly expressed (mean expression 17.2, SD 57.8 copies per cell).

Unsupervised hierarchical clustering of the miRNA expression profiles (Fig 4A) unambiguously separated the single cells into their original populations, consistent with the observed differential expression. However, these clustered expression profiles further revealed significant cell-to-cell heterogeneity within each cell line. For example, miR-221-3p expression was found to be bimodal in K562 cells, coinciding with both previous reports that miR-221-3p is down-regulated during erythropoiesis [39], and the propensity of K562 cells to undergo spontaneous erythroid differentiation in culture [40]. Similarly, the expression of miR-155-5p was bimodal in BaF3 cells, and this miRNA has been shown to play a key role in B lymphocyte maturation [41]. In contrast, the expression of miR-16-5p was not significantly different between the two populations (absolute median change 1.12×, p = 0.074, Wilcoxon rank-sum test), but was very heterogeneous within each population (K562: 0 to 28,622 copies per cell, CV of 342%; BaF3: 43 to 2,102 copies per cell, CV = 92%). miR-16-5p has been shown to be widely expressed amongst different tissue types from both human and rodents [42], and is known to regulate genes involved in cell cycle progression [43].

Collecting multiple measurements from the same single cell allowed us to use the observed variability to investigate miRNA co-expression networks. We identified 22 and 35 miRNA pairs that were significantly co-expressed in K562 and BaF3 cells, respectively (S4 Table). A subset of these pairs (14 of 43 unique significant pairs) was found in both populations, suggesting that they share a conserved regulatory framework. The three most highly co-expressed miRNA pairs in both populations occurred between miRNAs 17-5p, 20a-5p, and 92a-3p, with an average spearman correlation of 0.78 ± 0.12. These three miRNAs are all members of the miR-17~92 polycistronic cluster from which they are expressed as a single transcript prior to post-transcriptional processing into mature miRNAs [44, 45]. Despite their origins as a single primary transcript, we observed biased expression between individual miR-17~92 cluster members. For example, miR-17-5p was expressed at an average of 7.3 (SD = 1.2) and 10.3 (SD = 2.5) fold higher abundance than miR-20a-5p in K562 and BaF3 cells, respectively, an observation that implies a strong post-transcriptional regulatory mechanism. Indeed, the establishment of precise ratios between mature members of the miR-17~92 polycistron has been shown to be due to intrinsic characteristics conferred by secondary and tertiary structures of the primary 17~92 transcript [45].

Our single-cell expression measurements illustrate the danger in determining gene co-expression using bulk measurements. As described by Simpson’s paradox [46], an apparent trend can often be reversed or even disappear depending on how data are grouped. For example, if we naively remove the cell type classifier from our measurements of miR-24-3p and miR-92a-3p (Fig 4B, lower row), the genes are significantly negatively co-expressed (spearman’s correlation -0.37, p = 7.1×10^−5^), a conclusion that would also be made using bulk measurements. Exploiting the inherent cell-to-cell heterogeneity within a sample to identify truly co-regulated genes has been proposed as a solution to this problem [2], and multiplexed single-cell measurements are ideally suited to this task. Indeed, once the cells are correctly grouped by type, these two miRNAs are found to be significantly positively regulated (K562: spearman’s correlation 0.40, p = 6.1×10^−4^; BaF3: spearman’s correlation 0.44, p = 6.0×10^−4^).

We believe that streamlined technologies for highly multiplexed single cell RT-qPCR are particularly well suited to the investigation of cellular heterogeneity using miRNA signatures. Our measurements of twenty miRNAs in 176 single cells from two distinct cell types show clear evidence of well-known cell-to-cell heterogeneity while also recovering the established, evolutionarily conserved co-expression of the miR-17~92 polycistronic cluster. The broader investigation of miRNA expression at the single-cell level is anticipated to lead to exciting developments in miRNA and cell biology.

## Conclusion

We have presented a microfluidic device for performing highly multiplexed quantitative PCR on hundreds of single cells. Compared to existing single-cell RT-qPCR solutions, our device maintains the advantages of throughput, streamlined workflow, and reduced sample processing variations, labour, and reagent consumption seen in integrated devices, while adding the sought-after ability to collect multiple measurements per cell. Implementing this level of fluidic integration within a standard device footprint required a significant increase in feature density, which was implemented through combing high aspect ratio lithography, a reduction in the standard MSL valve size, and three-dimensional fabrication techniques. The resulting architecture can accommodate a variety of single-cell assays. Here we have demonstrated the quantitative measurement of mRNA and miRNA, but further assay development has the potential to expand into genotyping, epigenotyping, and combinations of assay classes. In terms of performance, we have established single-molecule sensitivity, a dynamic range of at least four orders of magnitude, a measurement precision of 15%, and high single-cell occupancy rates. The capabilities provided by this device fill an unmet need in the current repertoire of analytical methods for single-cell analysis by providing rapid measurements on panels of genes in hundreds of cells per run.

## Supporting information

S1 Fig Operation schematic for the two-step RT-qPCR workflow. (TIF)

S2 Fig Operation schematic for the one-step RT-qPCR workflow. (TIF)

S3 Fig Microfluidic device schematic. Schematic of microfluidic device for performing 200 20-plex single-cell RT-qPCR reactions. Features on the “flow” layer are indicated in blue, those on the “control” layer are indicated in red and interlayer connections are shown in orange. Scale bar 5 mm.
(TIF)

S4 Fig Measurement variability. Standard deviations from the measurements derived from the cell processing units (histogram) or the entire subarray (vertical lines) for each of three experiment replicates. There is a slight, but significant (mean s.d. = 0.125 to 0.179 for 200 and 0.2 pg/unit, respectively; p = 9.3×10^−18^, Kruskal-Wallis rank-sum test) shift in the distributions derived from the cell processing units, with those from lower RNA input amounts seeing higher variability. This shift, however, is much smaller than that seen between the variabilities calculated from the entire subarray (i.e. between vertical lines in figure) (mean s.d. = 0.156 to 0.579 for 200 and 0.2 pg/unit, respectively; p = 0.0273, Kruskal-Wallis rank-sum test). Furthermore, the difference in variability between the cell processing units and the full array is only significant between the two lowest concentrations (200 pg: p = 0.333, 20 pg: p = 0.264, 2 pg: p = 0.0105, 0.2 pg: p = 0.0105; Wilcoxon rank-sum test, Benjamini-Hochberg correction). We attribute this difference to the effects of stochastic sampling during RNA partitioning and initiation of cDNA synthesis.
(TIF)

S5 Fig Single-molecule cycle threshold cut-off. (A) Heatmap of unprocessed CT values used to calculate a cut-off cycle threshold value for a single cDNA molecule. (B) Histogram of unprocessed CT values with the calculated cut-off shown in red.
(TIF)

S6 Fig Variability of single-cell mRNA measurements. While not fully independent, replicate qPCR measurements (N = 3 for *RPPH1*, *GAPDH*, and *BCR-ABL*, N = 4 for *TBP* and *GGCX*) from each single cell (circles) give a measure of the qPCR variability within each cell-processing unit. In all cases, this variability is smaller than the expression variability between single cells (triangles).
(TIF)

S7 Fig Co-expression of endogenous control genes. Only modest co-expression is observed (average spearman correlation coefficient of 0.51, SD = 0.17). Spearman correlation coefficients, *r*_*s*_, and the corresponding co-expression significance are denoted; *** *p* < 0.001.
(TIF)

S8 Fig Differential miRNA expression. Boxplots show differential miRNA expression between K562 and BaF3 cells. Plots are sorted in order of decreasing significance, from top left to bottom right. Those in the bottom row were not significantly differentially expressed between the two populations. P-values were calculated using the Wilcoxon rank-sum test and Benjamini-Hochberg corrected.
(TIF)

S1 File AutoCAD drawing of the microfluidic device. (DWG)

S1 Table Single-cell gene expression method comparison. (PDF)

S2 Table Single-cell gene expression method performance comparison. (PDF)

S3 Table Single-molecule dilution detection measurements. Expected number of molecules and 95% confidence intervals based on the digital array response curve for a 52-chamber array. Cell processing units were counted as positive if more than 15 of the 20 detection chambers (75%) had a CT value less than the cut-off.
(PDF)

S4 Table miRNA co-expression significance. Spearman correlation coefficients, raw, and Benjamini-Hochberg corrected p-values for each pairwise comparison for the K562 and BaF3 cells. Pairs in which either cell population did not express both miRNAs are denoted with NA.
(XLSX)
