# Supplementary figures and images for: Highly multiplexed single-cell quantitative PCR
Source: PLoS One. 2018 Jan 29;13(1):e0191601. doi: 10.1371/journal.pone.0191601 (PMC5788347; doi:10.1371/journal.pone.0191601)

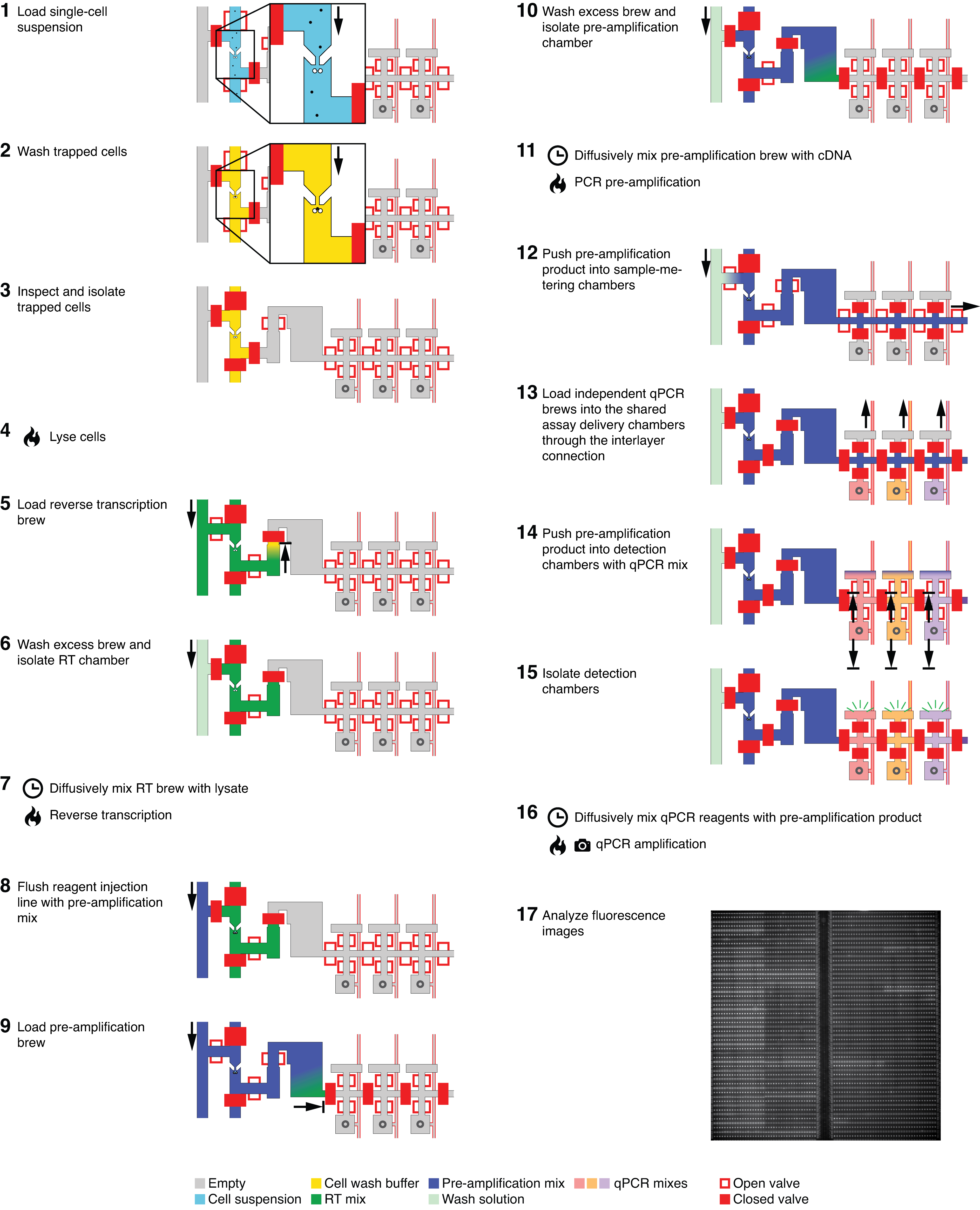

Supplement: S1 Fig — (TIF) [file pone.0191601.s001.tif]

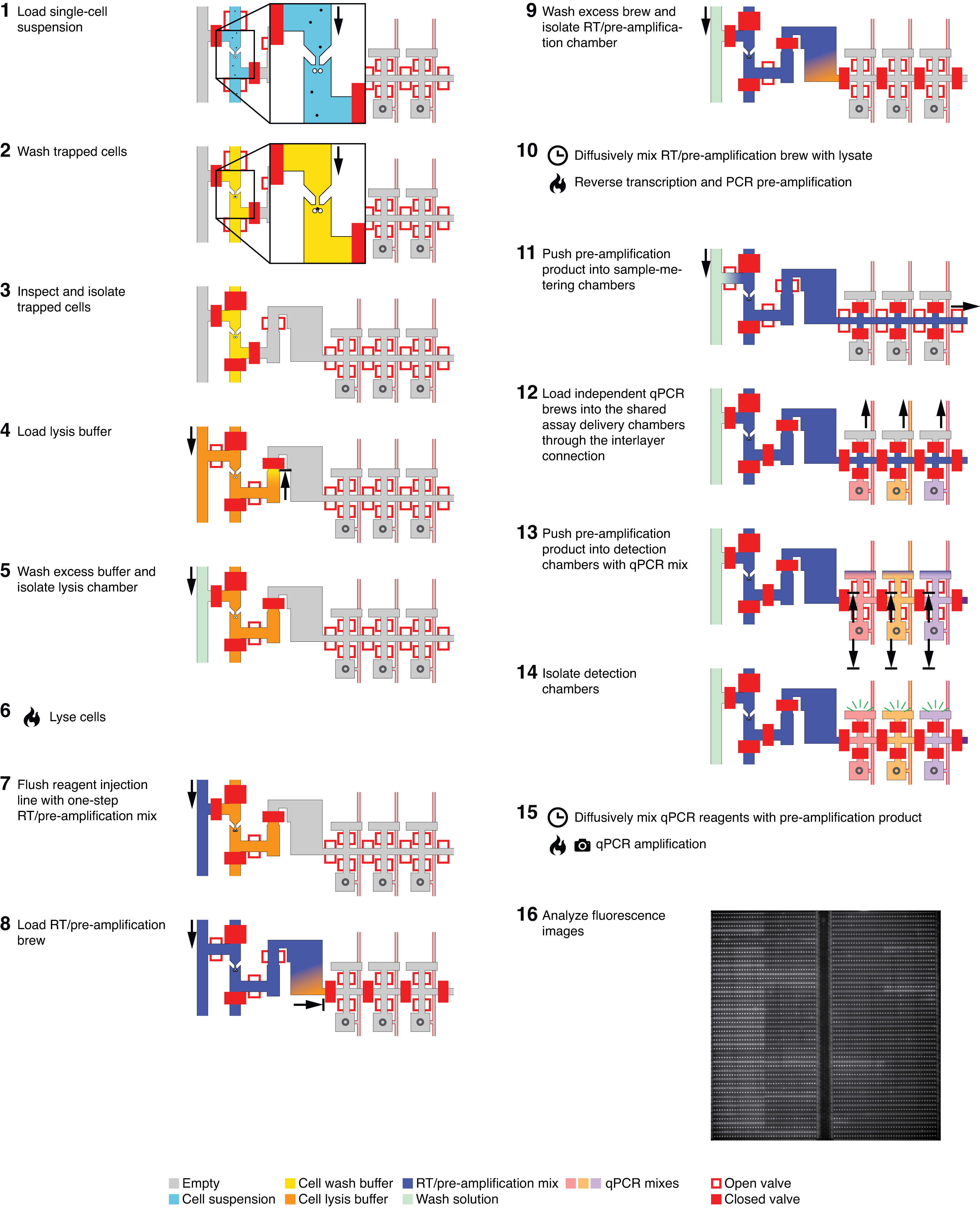

Supplement: S2 Fig — (TIF) [file pone.0191601.s002.tif]

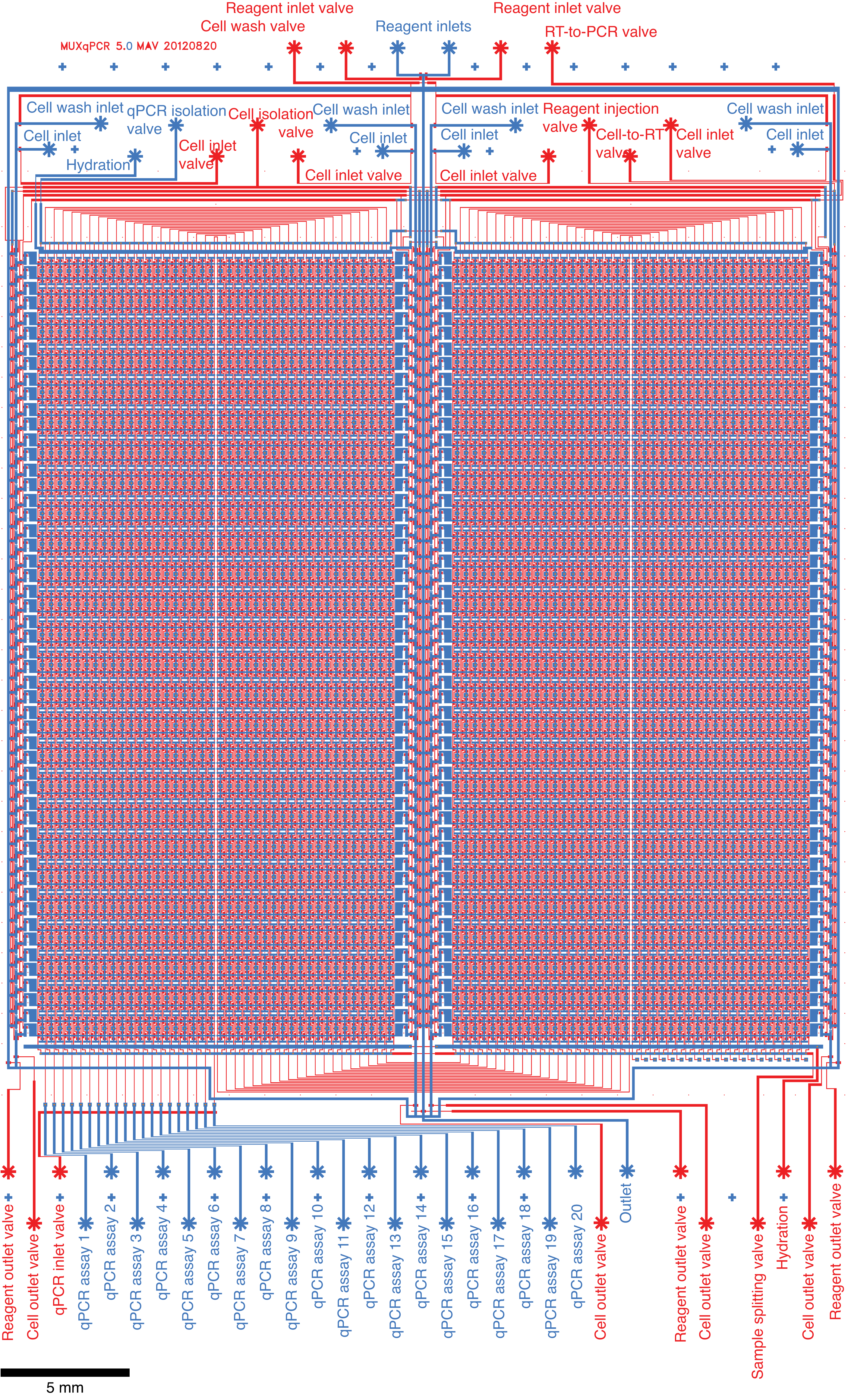

Supplement: S3 Fig — Schematic of microfluidic device for performing 200 20-plex single-cell RT-qPCR reactions. Features on the “flow” layer are indicated in blue, those on the “control” layer are indicated in red and interlayer connections are shown in orange. Scale bar 5 mm. (TIF) [file pone.0191601.s003.tif]

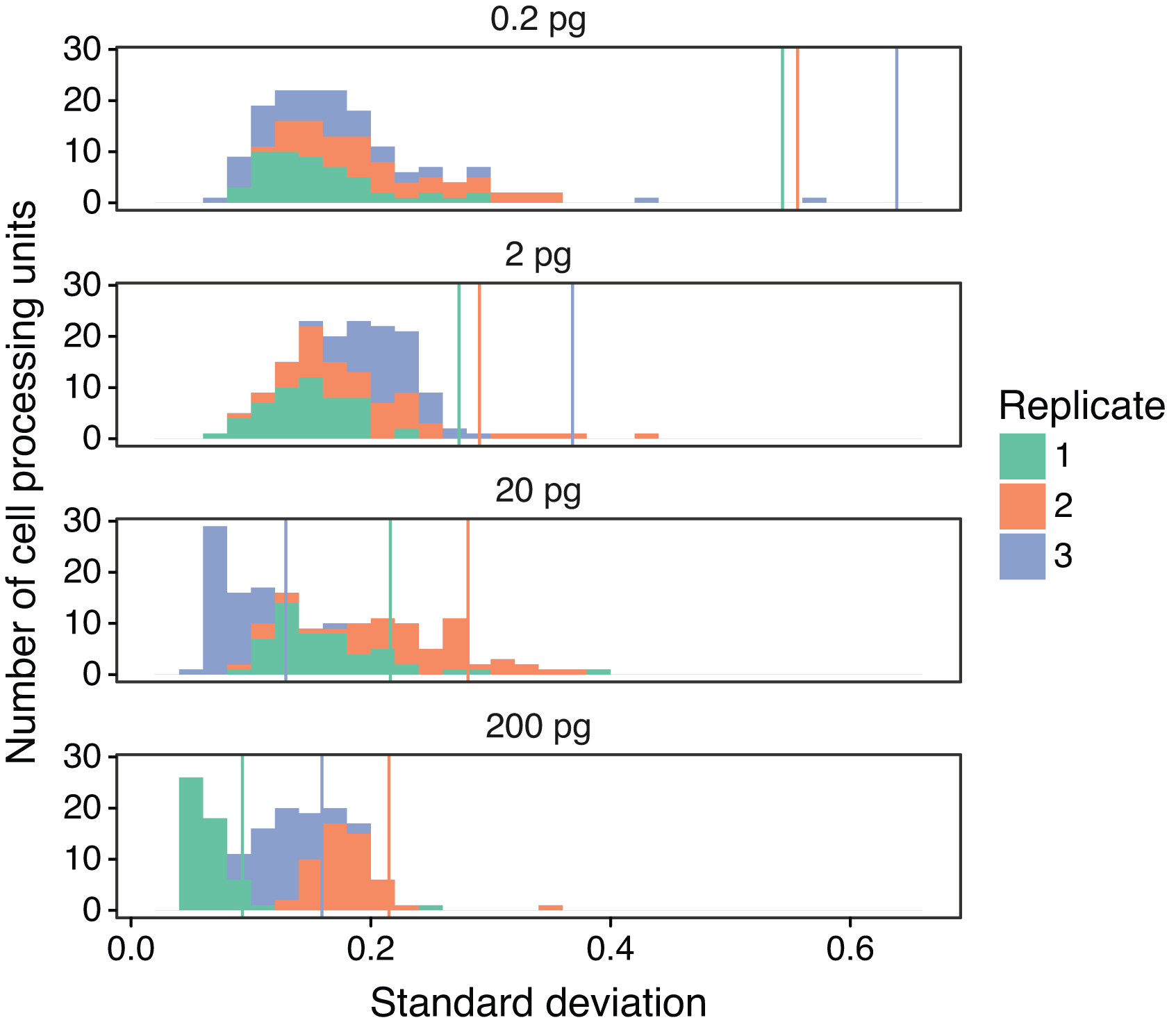

Supplement: S4 Fig — Standard deviations from the measurements derived from the cell processing units (histogram) or the entire subarray (vertical lines) for each of three experiment replicates. There is a slight, but significant (mean s.d. = 0.125 to 0.179 for 200 and 0.2 pg/unit, respectively; p = 9.3×10^−18^, Kruskal-Wallis rank-sum test) shift in the distributions derived from the cell processing units, with those from lower RNA input amounts seeing higher variability. This shift, however, is much smaller than that seen between the variabilities calculated from the entire subarray (i.e. between vertical lines in figure) (mean s.d. = 0.156 to 0.579 for 200 and 0.2 pg/unit, respectively; p = 0.0273, Kruskal-Wallis rank-sum test). Furthermore, the difference in variability between the cell processing units and the full array is only significant between the two lowest concentrations (200 pg: p = 0.333, 20 pg: p = 0.264, 2 pg: p = 0.0105, 0.2 pg: p = 0.0105; Wilcoxon rank-sum test, Benjamini-Hochberg correction). We attribute this difference to the effects of stochastic sampling during RNA partitioning and initiation of cDNA synthesis. (TIF) [file pone.0191601.s004.tif]

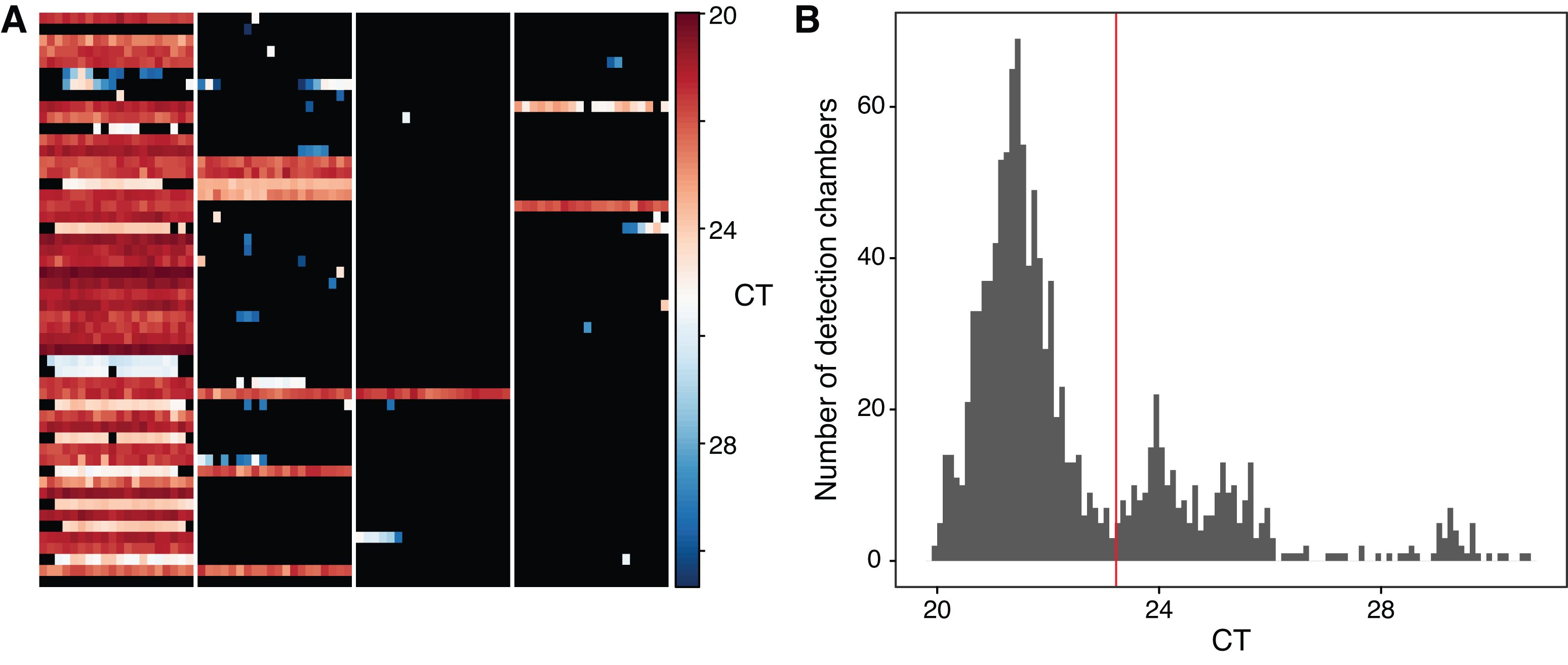

Supplement: S5 Fig — (A) Heatmap of unprocessed CT values used to calculate a cut-off cycle threshold value for a single cDNA molecule. (B) Histogram of unprocessed CT values with the calculated cut-off shown in red. (TIF) [file pone.0191601.s005.tif]

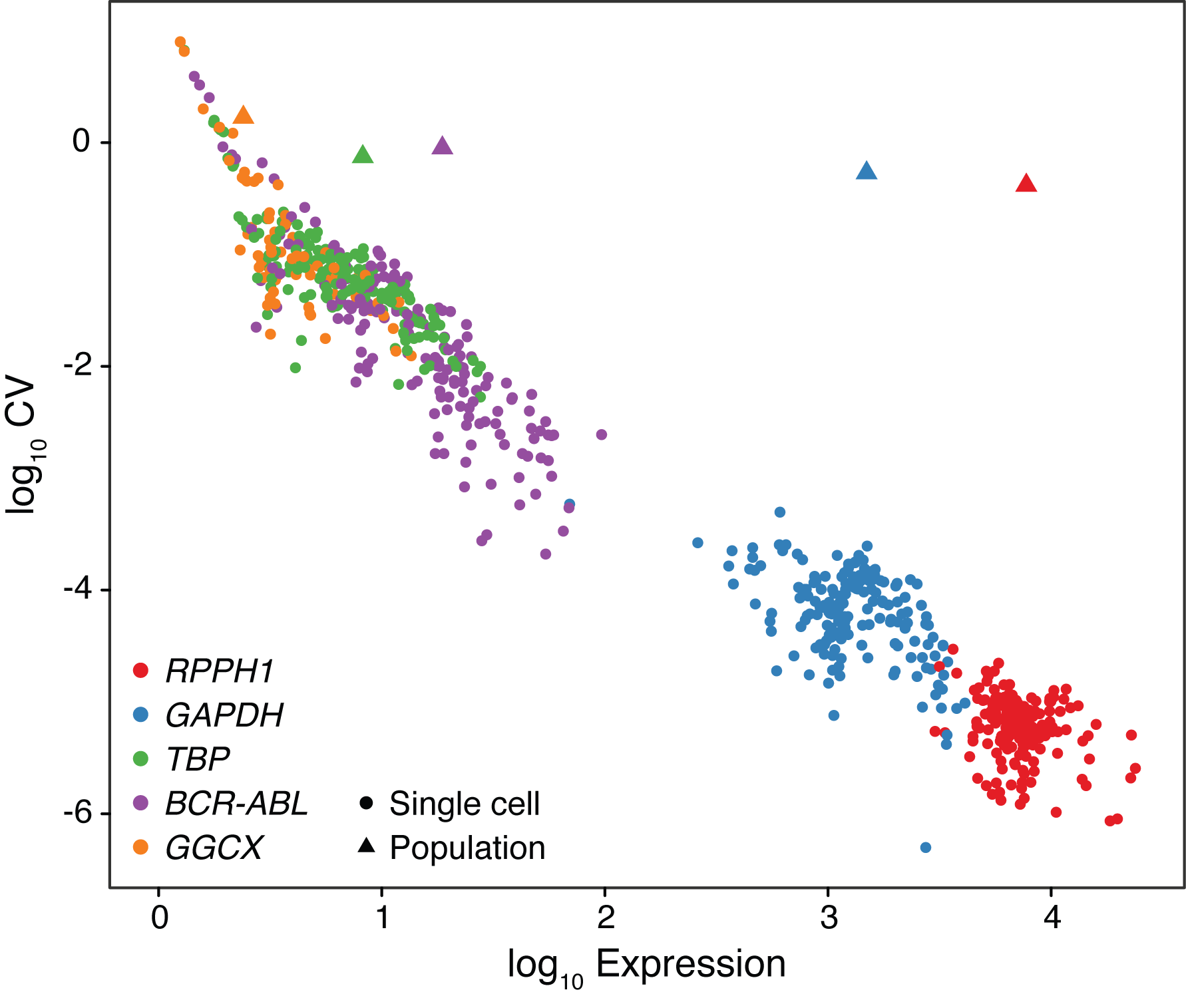

Supplement: S6 Fig — While not fully independent, replicate qPCR measurements (N = 3 for RPPH1, GAPDH, and BCR-ABL, N = 4 for TBP and GGCX) from each single cell (circles) give a measure of the qPCR variability within each cell-processing unit. In all cases, this variability is smaller than the expression variability between single cells (triangles). (TIF) [file pone.0191601.s006.tif]

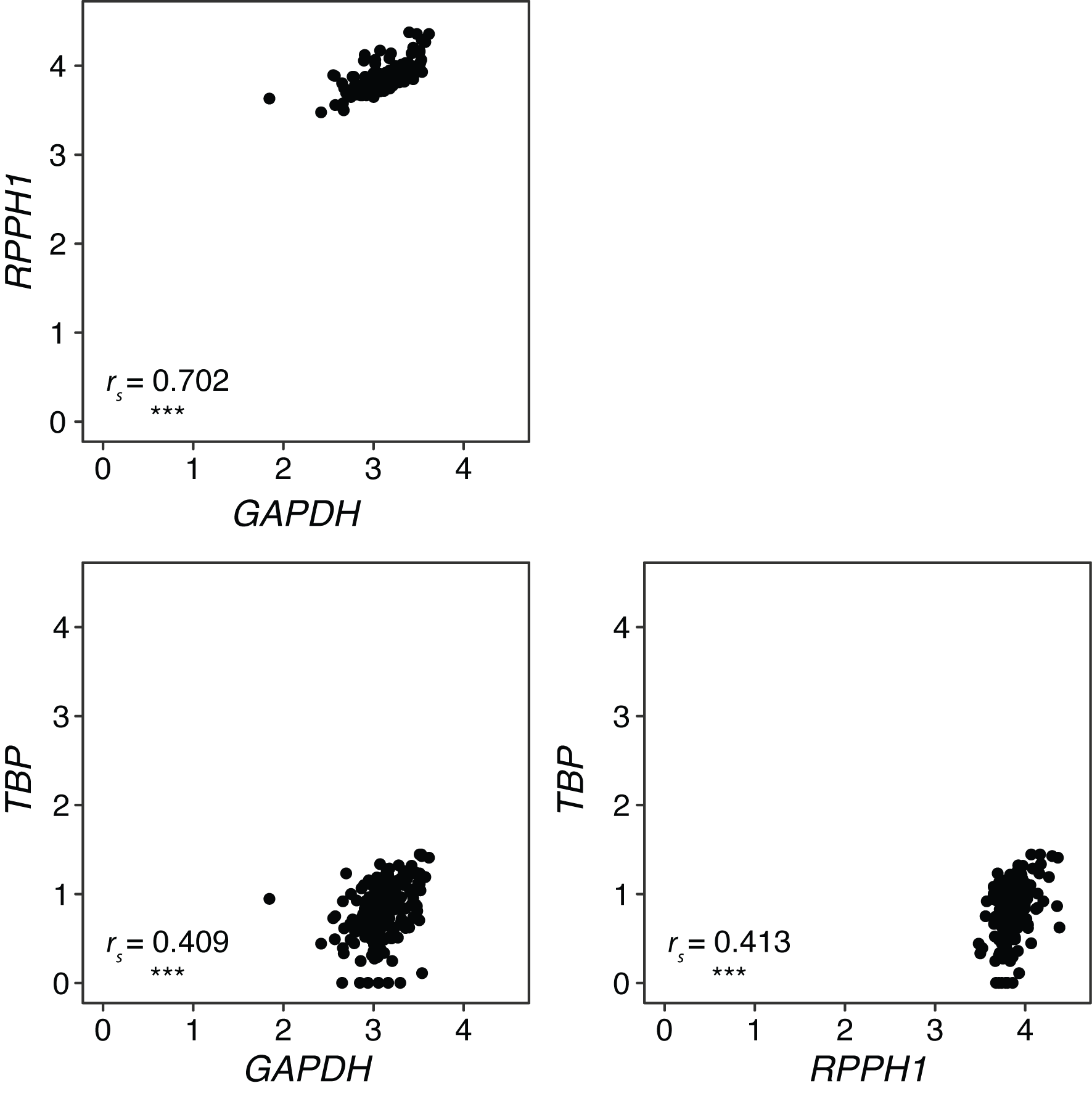

Supplement: S7 Fig — Only modest co-expression is observed (average spearman correlation coefficient of 0.51, SD = 0.17). Spearman correlation coefficients, rs, and the corresponding co-expression significance are denoted; *** p < 0.001. (TIF) [file pone.0191601.s007.tif]

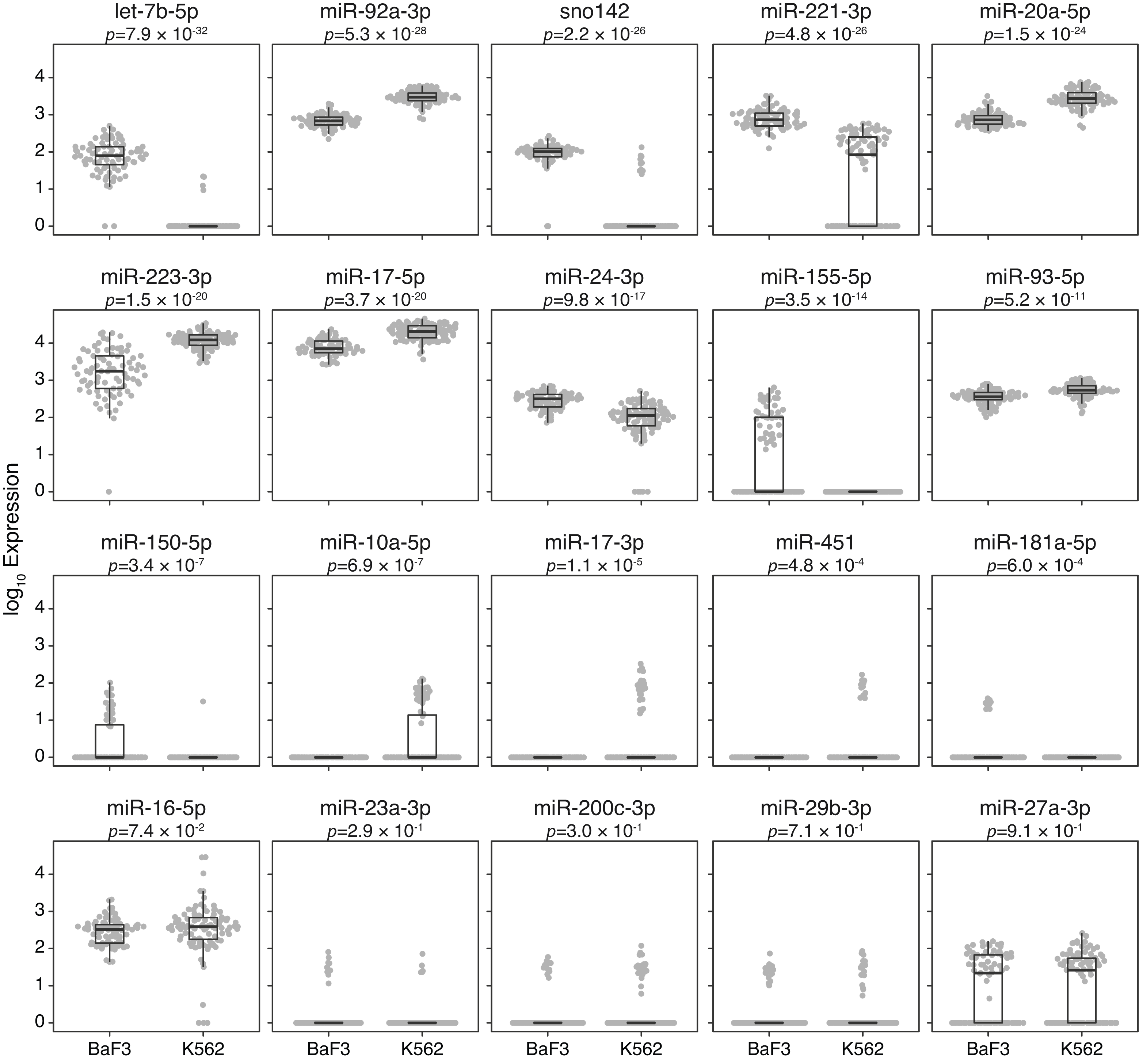

Supplement: S8 Fig — Boxplots show differential miRNA expression between K562 and BaF3 cells. Plots are sorted in order of decreasing significance, from top left to bottom right. Those in the bottom row were not significantly differentially expressed between the two populations. P-values were calculated using the Wilcoxon rank-sum test and Benjamini-Hochberg corrected. (TIF) [file pone.0191601.s008.tif]
