# Supplementary material for: Highly multiplexed single-cell quantitative PCR
Source: PLoS One. 2018 Jan 29;13(1):e0191601. doi: 10.1371/journal.pone.0191601 (PMC5788347; doi:10.1371/journal.pone.0191601)
Supplement: S1 Table — (PDF) [file pone.0191601.s010.pdf]

S1 Table. Single-cell gene expression method comparison.

Platform	Single-cell manipulation strategy	Detection method	Number of devices	Assayed molecule	Assay length	Cells per experiment	Assays per cell	Specialized equipment
This work	Integrated cell traps	qPCR	1	cDNA	6 hours	200	20-40	Microfluidic fabrication, device control, and qPCR
Single microfluidic systems	Integrated cell traps	qPCR, digital PCR	1	cDNA	2.5-4 hours	200-300	1-2	Microfluidic fabrication, device control, and qPCR or array scanner
Multiple microfluidic systems	Integrated cell traps	qPCR	2	cDNA	11 hours	96	48-96	Microfluidic chips, device control, and qPCR
Benchtop cell processing with microfluidic quantification	FACS, capillary mouth pipet	qPCR, digital PCR	1	cDNA	11 hours +	48-96	48-96	FACS, microfluidic chips, device control, and qPCR
Benchtop cell processing and quantification	FACS, capillary mouth pipet	qPCR	0	cDNA	11 hours +	~10-100	1-3	FACS
RNA-FISH	N/A	Molecule counting	N/A	RNA	~1 hour to overnight	~100	1-5, to 20	High resolution fluorescence microscope, probe sets
Multiplexed RNA-FISH	N/A	Molecule counting	N/A	RNA	~1.5 days	~100-40,000	100-1000	High resolution fluorescence microscope, fluid controllers, complex probe sets
