# Supplementary material for: Highly multiplexed single-cell quantitative PCR
Source: PLoS One. 2018 Jan 29;13(1):e0191601. doi: 10.1371/journal.pone.0191601 (PMC5788347; doi:10.1371/journal.pone.0191601)
Supplement: S2 Table — (PDF) [file pone.0191601.s011.pdf]

S2 Table. Single-cell gene expression method performance comparison.

Platform	Pre-amplification	Sensitivity	Measurement precision	Dynamic Range (orders of magnitude)	Example references
This work	12 PCR cycles	Single-molecule	+++	>4	
Single microfluidic systems	None	Single-molecule	+++	>4	[12, 14]
Multiple microfluidic systems	18-22 PCR cycles	Not investigated. Theoretical single-molecule	++	5.5	[20]
Benchtop cell processing with microfluidic quantification	18-22 PCR cycles	Not investigated. Theoretical limit depends on specific implementation.	++	5.5	[11, 13, 18, 19]
Benchtop cell processing and quantification	None	~10-100 molecules	+	~4	[9, 10]
RNA-FISH	None	Single-molecule	+++	>4	[15, 47-49]
Multiplexed RNA-FISH	None	Single-molecule	+++	>4	[16, 50]

Supplementary references

47. Shaffer SM, Wu M-T, Levesque MJ, Raj A. Turbo FISH: A Method for Rapid Single Molecule RNA FISH. PLOS ONE. 2013;8(9):e75120. doi: 10.1371/journal.pone.0075120.

48. Levesque MJ, Raj A. Single-chromosome transcriptional profiling reveals chromosomal gene expression regulation. Nature Methods. 2013;10:246. doi: 10.1038/nmeth.2372.

49. Levsky JM, Shenoy SM, Pezo RC, Singer RH. Single-Cell Gene Expression Profiling. Science. 2002;297(5582):836-40. doi: 10.1126/science.1072241.

50. Moffitt JR, Hao J, Wang G, Chen KH, Babcock HP, Zhuang X. High-throughput single-cell gene-expression profiling with multiplexed error-robust fluorescence in situ hybridization. Proceedings of the National Academy of Sciences. 2016;113(39):11046-51. doi: 10.1073/pnas.1612826113.
