# Supplementary material for: Highly multiplexed single-cell quantitative PCR
Source: PLoS One. 2018 Jan 29;13(1):e0191601. doi: 10.1371/journal.pone.0191601 (PMC5788347; doi:10.1371/journal.pone.0191601)
Supplement: S3 Table — Expected number of molecules and 95% confidence intervals based on the digital array response curve for a 52-chamber array. Cell processing units were counted as positive if more than 15 of the 20 detection chambers (75%) had a CT value less than the cut-off. (PDF) [file pone.0191601.s012.pdf]

S3 Table. Single-molecule dilution detection measurements. Expected number of molecules and 95% confidence intervals based on the digital array response curve for a 52-chamber array. Cell processing units were counted as positive if more than 15 of the 20 detection chambers (75%) had a CT value less than the cut-off.

Array	Dilution	Number of positive units	Expected number of molecules	Lower 95% confidence interval	Upper 95% confidence interval
1	1	36	60.7	48	79
2	1/5	5	5.3	4	7
3	1/25	1	1.0	1	1
4	1/125	1	1.0	1	1
